# A Memory-Efficient Deterministic Finite Automaton-Based Bit-Split String Matching Scheme Using Pattern Uniqueness in Deep Packet Inspection

**DOI:** 10.1371/journal.pone.0126517

**Published:** 2015-05-04

**Authors:** HyunJin Kim, Kang-Il Choi, Sang-Il Choi

**Affiliations:** 1 School of Electronics and Electrical Engineering, Dankook University, Yongin-si, Republic of Korea; 2 Advanced Communications Research Laboratory, Electronics and Telecommunications Research Institute, Daejeon, Republic of Korea; 3 Department of Applied Computer Engineering, Dankook University, Yongin-si, Republic of Korea; University of Catania, ITALY

## Abstract

This paper proposes a memory-efficient bit-split string matching scheme for deep packet inspection (DPI). When the number of target patterns becomes large, the memory requirements of the string matching engine become a critical issue. The proposed string matching scheme reduces the memory requirements using the uniqueness of the target patterns in the deterministic finite automaton (DFA)-based bit-split string matching. The pattern grouping extracts a set of unique patterns from the target patterns. In the set of unique patterns, a pattern is not the suffix of any other patterns. Therefore, in the DFA constructed with the set of unique patterns, when only one pattern can be matched in an output state. In the bit-split string matching, multiple finite-state machine (FSM) tiles with several input bit groups are adopted in order to reduce the number of stored state transitions. However, the memory requirements for storing the matching vectors can be large because each bit in the matching vector is used to identify whether its own pattern is matched or not. In our research, the proposed pattern grouping is applied to the multiple FSM tiles in the bit-split string matching. For the set of unique patterns, the memory-based bit-split string matching engine stores only the pattern match index for each state to indicate the match with its own unique pattern. Therefore, the memory requirements are significantly decreased by not storing the matching vectors in the string matchers for the set of unique patterns. The experimental results show that the proposed string matching scheme can reduce the storage cost significantly compared to the previous bit-split string matching methods.

## Introduction

Nowadays, one of the most powerful methods of ensuring network security and quality of service (QoS) is DPI, in which the payloads are analyzed to determine whether target patterns are matched or not in the application layer. In many cases, target patterns are composed of multiple characters; therefore, the string matching engine is an essential component of modern DPI [1]. With the advance of networking services, the number of target patterns increases and the length of the pattern, which means the number of characters in it, can vary dramatically.

In order to overcome the problem of diverse pattern lengths, DFA-based string matching approaches have been developed. Even though there have been several recent attempts to reduce the memory requirements in field programmable gate array (FPGA) devices in [2–4] and ternary content-addressable memory (TCAM) in [5], DFA-based string matching using general or static memory blocks can provide several advantages; firstly, the deterministic transition step is achieved between states, regardless of the input symbols. Secondly, the number of output transitions from a state can be fixed. Therefore, both the regularity and scalability can be guaranteed in DFA-based string matching. Memory-based string matching with distributed block memory is simple to implement. As shown in [6], if a redundant memory block is provided, the updatability can be increased. Even though it has several advantages, as mentioned above, sparse memory usage is one of main problems in DFA-based string matching.

In the Aho-Corasick algorithm described in [7], the number of state pointers in each state is 256 for ASCII (8 bits) input, which imposes a significant burden on the memory requirements of the DFA-based string matching engine. Bit-split string matching reduces the number of state pointers using multiple FSM tiles with several input bit groups [6]. In the bit-split string matching engines described in [6], the output state should have a partial matching vector (PMV); the full matching vector (FMV) is obtained by performing a bitwise AND operation between the PMVs from the FSM tiles, where each bit in the FMV represents whether its own pattern is matched or not. Because the memory requirements for storing PMVs are proportional to the numbers of states and patterns to be mapped in a string matcher, several previous string matching schemes reduce the memory requirements by sharing PMVs [8, 9]. In [8], a separate PMV table is adopted for each FSM tile, where the memory requirements of the PMV table is proportional to the square number of patterns in a string matcher. In [9], PMVs are shared between FSM tiles in the bit-split string matcher. Therefore, only one PMV table is adopted in a string matcher to reduce the memory requirements. In [10], considering pattern lengths, heterogeneous string matchers with different maximum number of states and patterns to be mapped are adopted. However, many memory blocks for storing PMVs are still required in [8–10].

When multiple patterns are matched in a state of the bit-split DFA, the multiple patterns are elements in the set with *non-unique* patterns. For example, let us assume that there are four patterns {“abcd,”, “cd,”, “d,” “fg”} in a set of patterns. If a pattern “abcd” is matched, patterns “cd” and “d” can be matched simultaneously in each FSM tile. Because these three patterns are not unique, the four patterns can be elements of the set with non-unique patterns. For a set with non-unique patterns, the bit-split string matching techniques with PMVs such as those described in [6, 8] can be applied to recognize which patterns are matched simultaneously. For a set of *unique* patterns, only the unique matching index for a matched pattern is stored for each output state, which reduces the memory requirements by not storing the PMVs.

This paper proposes a memory-efficient DFA-based string matching scheme that reduces the memory requirements by not storing the matching vectors. In the proposed scheme, the proposed pattern grouping divides a set of all target patterns into the set with non-unique patterns and set of unique patterns. For the set with non-unique patterns, the bit-split string matching technique with PMVs is applied. On the other hand, in the DFA for the set of unique patterns, only a single pattern is matched in each state. For the set of unique patterns, because the bit-split string matchers store only the pattern matching index, the memory requirements can be reduced by not storing the PMVs. The experimental results show that the proposed scheme can be effective in reducing the memory requirements. In the experiments, the Snort [11] and ClamAV [12] rule sets are adopted. In addition, several statistically generated rule sets are used to analyze the pattern uniqueness. The proposed scheme can be applied to other bit-split DFA-based string matching architectures and pattern mapping approaches such as those described in [13–15].

This paper is organized as follows: firstly, previous works are reviewed to show the effectiveness of the bit-split string matching. Then, the background of this paper is explained in detail. In the section of background, several definitions and lemmas are provided to clarify the proposed scheme. In addition, the pattern uniqueness in the bit-split string matching is explained. In the following section, the parallel memory-based bit-split string matching architecture for the set of unique patterns and set with non-unique patterns is described. Then, the proposed pattern grouping and mapping algorithms for obtaining the set of unique patterns and mapping target patterns are explained. Finally, the experimental environments and results are provided. In addition, the analysis of the pattern uniqueness and practical implementation issue are discussed.

## Previous Works

In the survey of [1], string matching schemes were classified into three types: automaton-based, heuristic-based, and filtering-based string matching schemes. A heuristic-based string matching scheme can accelerate the search by skipping characters not in a match. One of well-known heuristic-based string matching schemes is the Boyer-Moore algorithm [16]. By performing comparisons at different alignments of a pattern and text to be searched, occurrences of the pattern can be searched. With the information obtained by preprocessing the pattern, many alignments can be skipped. However, in the worst case, there are no skipped characters. When the pattern length and number of characters in a text are assumed as *m* and *n*, the time complexity can be *O*(*nm*) in the worst case. Therefore, as shown in [1], heuristic-based string matching scheme is not suitable in DPI because algorithmic attacks can degrade overall performance in the string matching engine. Even though the string matching with multiple patterns can be possible in heuristic-based string matching [17], multiple processing elements or cores should be equipped. Therefore, it is expected that heuristic-based scheme is not effective for a large number of target patterns.

Due to the parallelized string matching with multiple patterns, automaton-based or filtering-based string matching schemes are preferred. Filtering-based string matching scheme adopts hashing [18] or bloom filter [19], which are memory-efficient in processing bit vectors. The filtering-based string matching scheme can quickly exclude input data that do not contain patterns to be matched. Because the efficiency of the filtering-based scheme is based on the assumption that patterns are rarely matched in payloads, the filtering-based string matching scheme might suffer from algorithmic attacks in the worst case scenario.

On the other hand, in automaton-based string matching scheme, multiple patterns are mapped using states and state transitions between states. Especially, DFA-based string matching scheme performs a fixed number of state transitions at a time. Therefore, linear worst-case performance can be guaranteed. In addition, target patterns crossing multiple payloads can be matched because each state contains the information of input sequence. However, the DFA-based string matching scheme has large memory requirements to store the information of states and state transitions for each state. The Aho-Corasick algorithm was proposed for bibliographic string searches with compressed DFAs [7]. In the Aho-Corasick algorithm, a DFA should contain failure pointers from each state to the longest suffix state or matched subpatterns. By sharing common prefixes, the number of states in a DFA is reduced; therefore, the information of states can be compressed. In the traditional Aho-Corasick algorithm, however, due to the large number of state transitions (e.g. 256 for the input symbol of ASCII character code), the memory requirements for storing state transitions in each state are great.

Automaton-based string matching scheme can be implemented using general memory. For example, two-dimensional memory architecture is adopted in the implementation of the Aho-Corasick algorithm. A memory-based string matching engine can have both updatability and flexibility because memory contents are easily updated with a memory interface. Due to the large memory requirements for storing state transitions, several researches have been studied to reduce the number of stored state transitions. In [2, 20, 21], by adopting configurable logics and distributed memory in an FPGA, only the state transitions towards non-initial states are stored. However, compared to the memory-based string matching engine, the flexibility and updatability in an FPGA cannot be sufficient. In [5, 22], TCAM is adopted for compressing the information of state transitions in a DFA-based string matching. However, due to the high price and power consumption of TCAM, the usage of TCAM in the DFA-based string matching can be limited.

In order to reduce the memory requirements for storing state transitions, bit-split DFA-based string matching was proposed in [6]. By splitting one or more than one ASCII character code into several bit groups, multiple DFAs are constructed for each input bit group, so that the total number of state transitions in each state could be reduced. In addition, multiple homogeneous string matchers are operated in parallel, where a small number of target patterns are mapped onto each string matcher. Therefore, the regularity can be guaranteed in the implementation. In addition, by changing memory contents using a spare string matcher in real time, high updatability can be achieved. In order to identify matched target patterns in a state, each state should contain its own match vector with a set of bits or PMV, where the value of each bit indicates whether the related target patterns are matched or not in the state. Therefore, the memory requirements for storing match vectors might be significant.

Several bit-split string matching schemes were developed to reduce memory requirements. However, several works are related only to the pattern sorting based on the original bit-split string matching. The original bit-split string matching in [6] increases the number of shared states in each string matcher using the lexicographical pattern sorting. The variety of target pattern lengths, however, causes unbalanced memory usage between string matchers because the number of mapped target patterns onto each string matcher could vary. In addition, our several previous researches focus on only the pattern sorting that decides the order of patterns to be mapped onto a string matcher [14, 15, 23]. In these researches, by balancing memory usage between string matchers, memory requirements are significantly reduced. However, the problem of large memory requirements for storing match vectors is not considered.

In the previous works in [6, 13, 24, 25], several architectures based on the bit-split string matching scheme were proposed. In [13, 24], the memory requirements for storing state transitions towards initial states are reduced. In [6, 25], a multi-byte string matching is performed by multiplying the number of memory blocks. Even though the previous works related to the pattern grouping and architectures mentioned above have developed new bit-split string matching schemes, a string matcher should have memory blocks for storing match vectors, which can be the disadvantage in the new architectures based on the bit-split string matching scheme.

Several approaches to reduce the memory requirements for storing match vectors were proposed. In [10], the architecture with heterogeneous string matchers is adopted to enhance the efficiency of memory usage for mapping target patterns with various lengths. In [10], for the patterns with short pattern lengths, the FSM tile with a small number of states and PMVs is adopted. On the other hand, for the patterns with long pattern lengths, a large number of states and PMVs is adopted. By increasing the number of patterns to be mapped in the string matchers for short target patterns, the number of bits in a PMV for long target patterns can be reduced in the string matchers. However, if the number of patterns with short pattern lengths is not large, the reduced memory requirements are not sufficient. In [8], the memory requirements for storing match vectors are reduced by relabeling states and eliminating the match vectors of non-output states. However, each FSM tile has its own match vector table in order to share match vectors between states, which can increase the number of memory block cells. In [9], multiple FSM tiles share the same match vector table in order to minimize the memory requirements. However, multiple memory accesses should be serially performed by obtaining multiple match vectors in a match vector table, which degrades overall performance. In addition, the previous studies cannot eliminate the memory requirements for storing match vectors in a string matcher.

## Background

In this section, the description of the pattern uniqueness based on the ASCII character input is shown. Then, the concept of the pattern uniqueness in the bit-split string matching is explained with several examples.

### Non-unique and unique patterns

In the DFA-based string matching technique, the information used for representing which patterns are matched should be provided for each state. In this case, the memory requirements for storing the information can depend on the uniqueness of the patterns in a set. A set of target patterns can be divided into the set with non-unique patterns and set of unique patterns. A non-unique pattern is defined as follows:

**Definition 1**. *For the sequence of input symbols, the non-unique pattern can be the suffix of other patterns, or other patterns can be the suffixes of the non-unique pattern in the set with non-unique patterns*.

In the example of an ASCII input sequence, Let us assume that there are four patterns in the set with non-unique patterns {“abcd,”, “cd,”, “d”, “fg”}. In the example, patterns “cd” and “d” are suffixes of a pattern “abcd.” In the set with non-unique patterns, it is possible that not all of patterns are non-unique. However, multiple patterns can be matched at the same time for the set with non-unique patterns because a pattern and its suffix are matched simultaneously. Lemma 1 is provided to show the characteristics of the pattern matching identification for the set with non-unique patterns:

**Lemma 1**. *When the number of patterns in the set with non-unique patterns is N, the number of bits required for representing which patterns are matched is N.*

*Proof*. Let us assume that its related pattern is matched when a bit for the pattern matching identification is one. When *N* patterns are elements of the set with non-unique patterns, it is possible that a pattern can be the suffix of the other *N* − 1 patterns in a state. For the state mentioned above, *N* bits are required for *N* patterns in order to identify that all patterns are matched in the state. Therefore, *N* bits are required to represent which patterns are matched.

In the example of four patterns mentioned above, the number of required bits for the pattern matching identification can be four. Considering Lemma 1, as the number of patterns in the set with non-unique patterns increases, the memory requirements for the pattern matching identification in a state increase proportionally to the number of patterns. On the other hand, the unique pattern is defined as follows:

**Definition 2**. *For the sequence of input symbols, the unique pattern is not the suffix of any other pattern, and the other patterns are not suffixes of the unique pattern in the set of unique patterns*.

Considering Definition 2 of the unique pattern, there is only one pattern matched in a state for the set of unique patterns. Therefore, there is no need to adopt multiple bits to represent which patterns are matched. For the set with non-unique patterns, each bit is used for its related pattern matching identification. On the other hand, for the set of the unique patterns, only the binary index is required to represent which pattern is matched for a state. If there are *N* unique patterns in a set, the size of the binary index is *log*_2_ *N*.

### Grouping for obtaining a set of unique patterns

**Fig 1:**
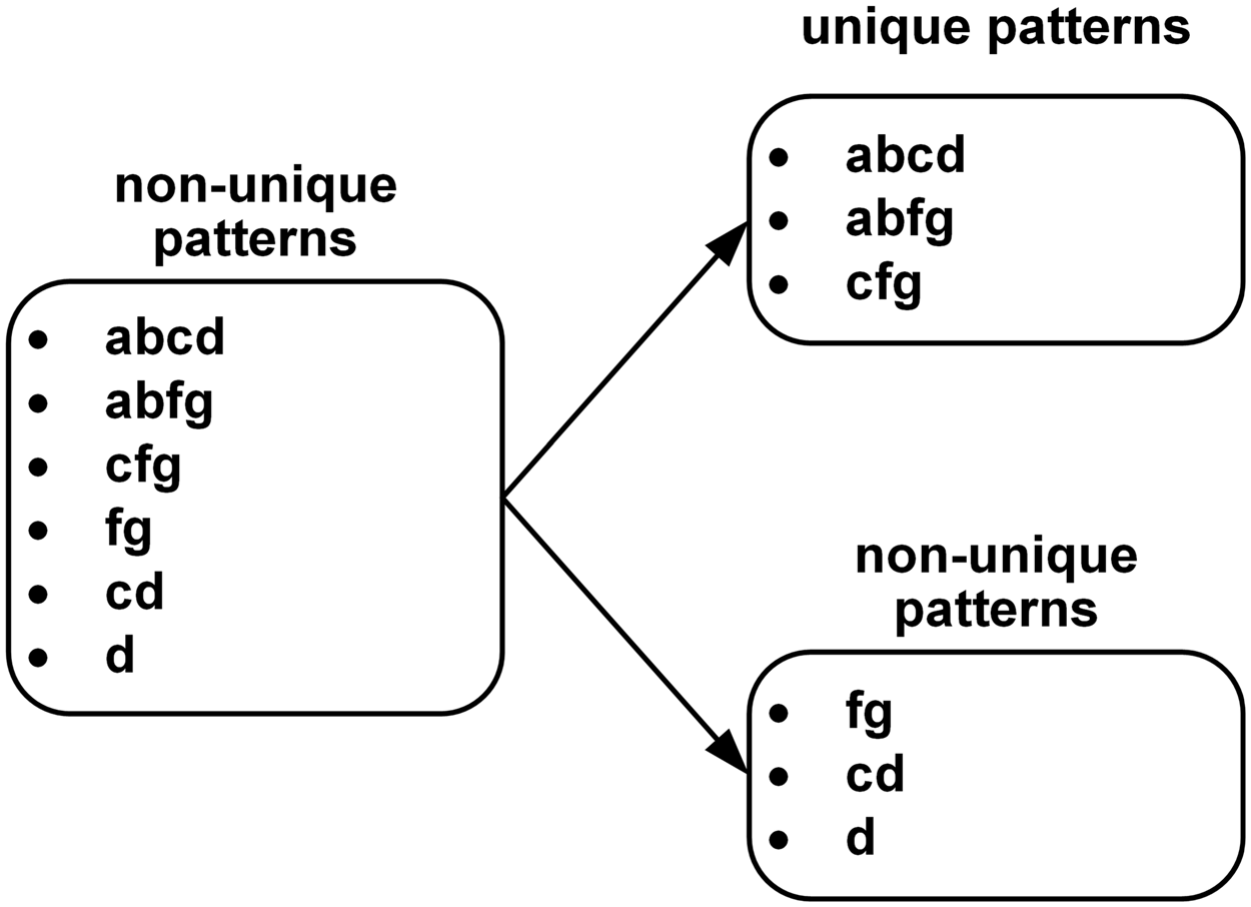
Example of pattern grouping.

Initially, a set of all target patterns could be the set of non-unique patterns, because a pattern can be suffix of any other target pattern in the set of all target patterns. In order to obtain a set of unique patterns, all of the target patterns can be grouped into two sets by considering the uniqueness of patterns. Fig 1 shows an example of pattern grouping for six patterns.

Considering Definition 1, the original set of target patterns is for the non-unique patterns. For example, patterns “fg” and “cd” are the suffixes of patterns “abfg” and “abcd,” respectively. In this case, if patterns {“fg,” “cd,” “d”} are excluded from the original set, a set of patterns {“abcd,” “abfg,” “cfg”} can be the set of the unique patterns. These excluded patterns can be the elements of the set with non-unique patterns because “d” is the suffix of “cd.” In other words, patterns “d” and “cd” are non-unique patterns.

**Fig 2:**
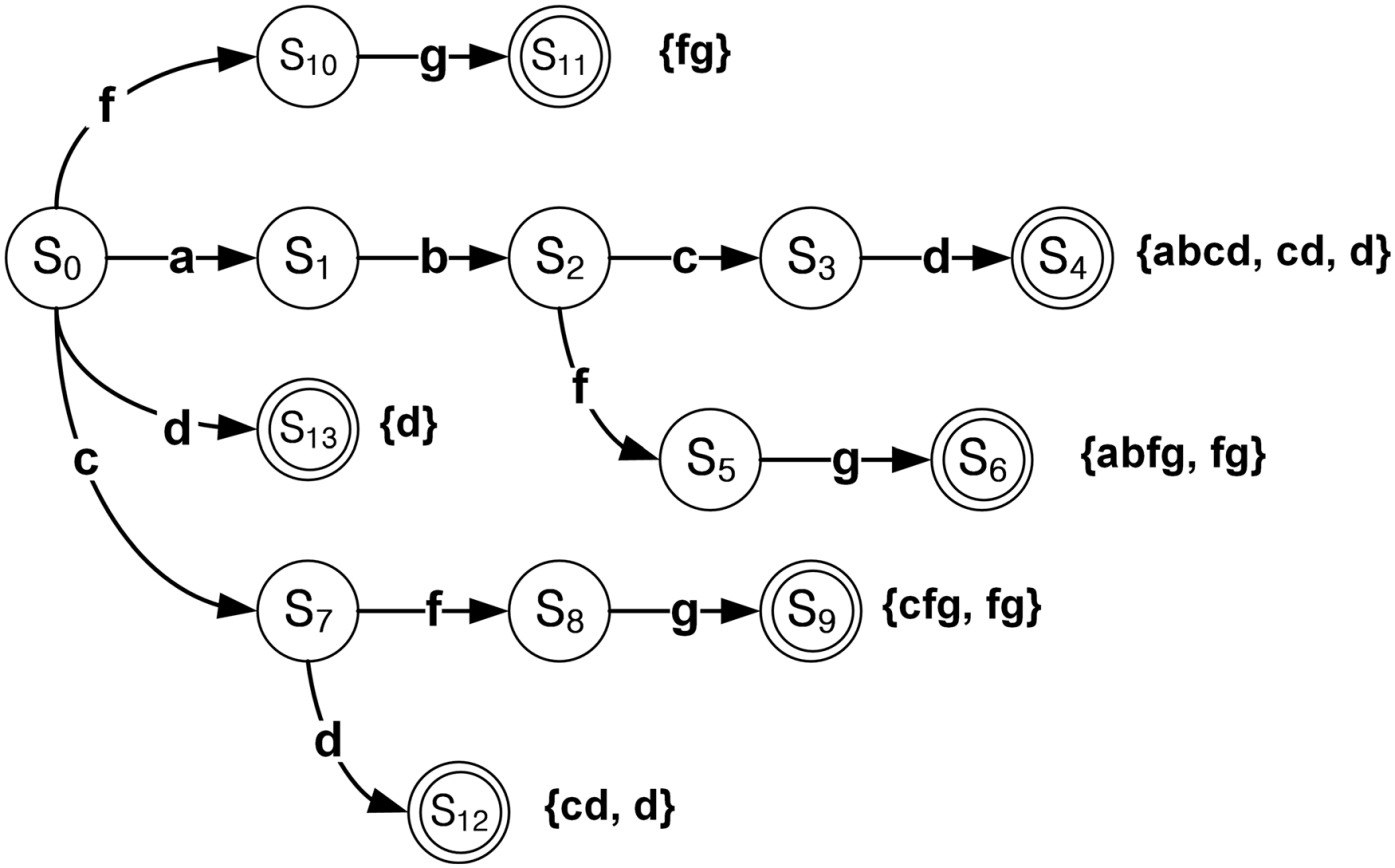
Example of DFA for all of target patterns in Fig 1.

Fig 2 shows an example of the DFA for the original six target patterns in Fig 1, where the failing pointers are not shown for clarity. The arrow means the state transition according to the input symbol shown on the arrow. State *S*_0_ is the initial state. In addition, the double-circled states *S*_4_, *S*_6_, *S*_9_, *S*_11_, *S*_12_, and *S*_13_ are the output states, where their related patterns in the curly brackets are matched in the output states, respectively. Multiple patterns are matched in *S*_4_, *S*_6_, *S*_9_, and *S*_12_. Therefore, when the DFA is constructed with the six target patterns, the patterns are the elements of the set with non-unique patterns.

**Fig 3:**
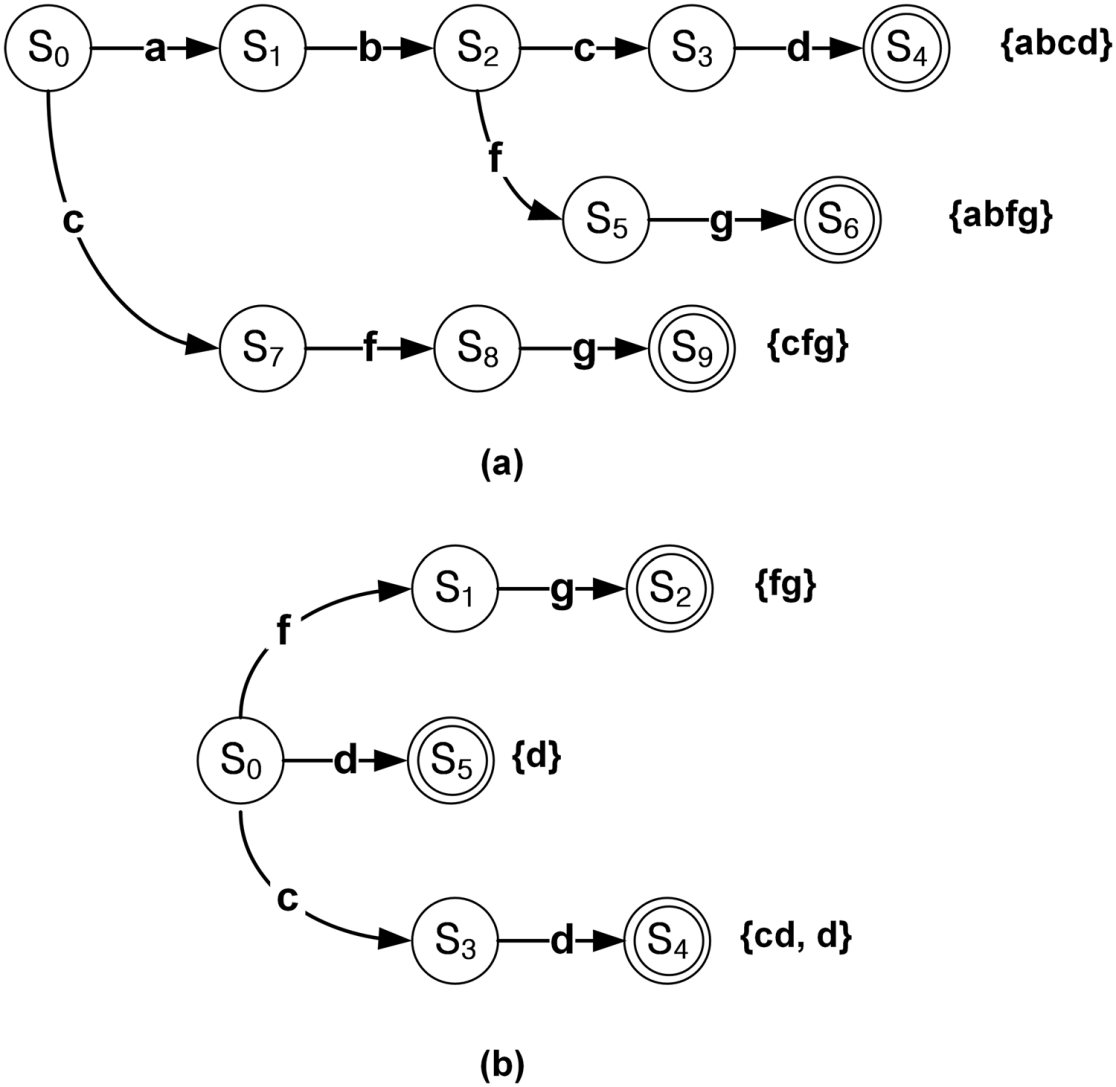
Example of DFAs after considering the pattern uniqueness. (a) DFA for the set of unique patterns; (b) DFA for the set with non-unique patterns.

After grouping the target patterns, the set of non-unique patterns and set with non-unique patterns are obtained. Fig 3 shows an example of the DFAs for the obtained two sets. Unlike the DFA in Fig 2, only one pattern is matched in the output state in Fig 3(a), which means the DFA for the set of unique patterns. On the other hand, multiple patterns can be matched in the output state *S*_4_ for the set with non-unique patterns, as shown in Fig 3(b).

### Bit-split string matching using pattern uniqueness

**Fig 4:**
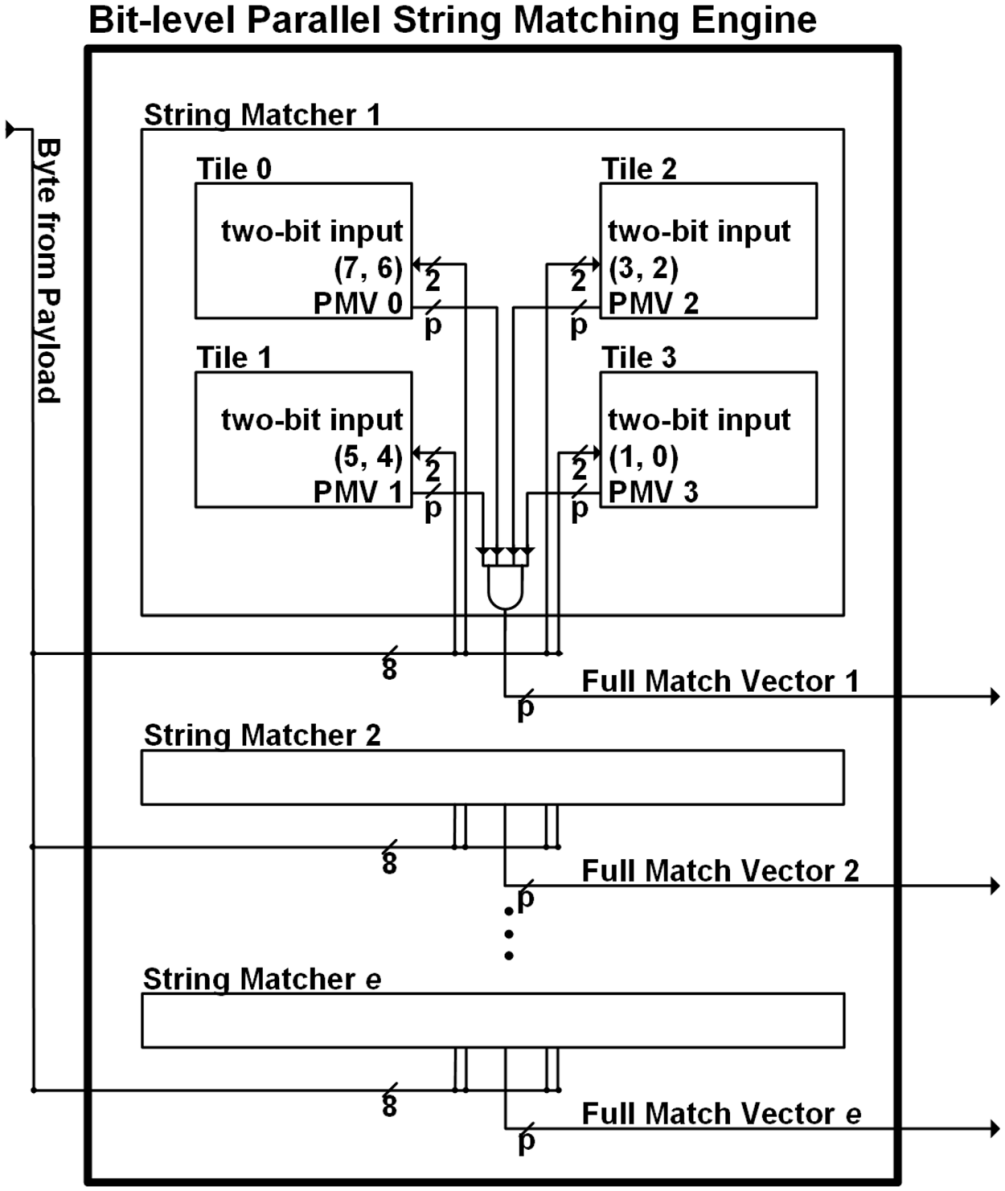
Bit-split string matching engine.

In order to reduce the hardware cost in the DFA-based string matching engine, the bit-split string matching engine based on the Aho-Corasick algorithm [7] was proposed in [6]. Fig 4 shows the architecture of the bit-split string matching engine described in [6]. By splitting one ASCII character code into several bit groups, multiple FSM tiles are constructed for each input bit group in order to reduce the number of state transitions in each state. In Fig 4, two bits are inputted for each FSM tile, where the number in parentheses refers to the bit position of an input symbol from the LSB (least significant bit) in the ASCII code. In addition, the target patterns are lexicographically grouped and then each set of the grouped target patterns is mapped onto one homogeneous string matcher.

The pattern identification in the multiple FSM tiles of a string matcher requires that each state contains the match vectors for the target patterns mapped on the string matcher. As shown in Fig 4, by performing a bitwise AND operation between the PMVs from the FSM tiles, the FMV is obtained. Each bit within it represents whether its related pattern is matched or not. Because the number of bits in a PMV is proportional to the number of patterns, the memory requirements for storing the PMVs might be significant.

Because the target patterns are mapped onto multiple FSM tiles, the pattern grouping for the bit-split string matching considers all cases for the input bit groups. The unique pattern of the bit-split string matching is defined in Definition 3.

**Definition 3**. *In the set of unique patterns of the bit-split string matching, the unique pattern is not the suffix of any other pattern, and the other patterns are not the suffixes of the unique pattern for any of the DFAs with input bit groups*.

Lemma 2 is provided in order to show the characteristics of the pattern uniqueness for the bit-split string matching.

**Lemma 2**. *For the DFAs for the unique patterns of the bit-split string matching, there is no need to adopt the match vector*.

*Proof*. In the DFA with each bit group, only one pattern is matched in an output state. Therefore, for the unique patterns of the bit-split string matching, only one pattern can be matched at a time, thus allowing the match vector for the unique patterns of the bit-split string matching to be removed.

Considering Lemma 2, the uniqueness in the bit-split string matching is different from that of the traditional DFA with the ASCII input sequence. The example of three patterns “abcd,” “abfg,” and “cfg” in Fig 3 is based on ASCII or 8-bit input; therefore, the patterns are unique when ASCII code input is assumed. However, the patterns are not unique in the bit-split architecture with several input bit groups. In order to explain the pattern uniqueness in the bit-split string matching architecture, another example for the bit-split architecture with four FSM tiles has been added. The ASCII binary codes of characters ‘a,’ ‘b,’ ‘c,’ ‘d,’ ‘f,’ ‘g,’ and ‘h’ are shown in Table 1.

**Table 1:** ASCII binary codes.

char.	value	char.	value
a	01100001_2_	b	01100010_2_
c	01100011_2_	d	01100100_2_
e	01100101_2_	f	01100110_2_
g	01100111_2_	h	01101000_2_

For the bit-split string matching architecture, two input bits are adopted for each FSM tile. In the example, the *i*-th bit from the LSB is grouped with the (7 − *i*)-th bit in order to balance the change in each bit position, as shown in [13]. Table 2 shows multiple bit-split patterns with two-bit vectors for patterns “abcd,” “abfg,” and “cfg,” where *pattern*_*i*, *j*_ means that the *i*-th and *j*-th bits are adopted in the pattern with two-bit vectors. In addition, the right arrow, →, means the sequence of two-bit vectors. As shown in Table 2, *pattern*_4,3_ for patterns “abcd” and “abfg” is the same. Therefore, for the bit-split string matching with two-bit input, patterns “abcd” and “abfg” are not unique. In addition, *pattern*_4,3_ of pattern “cfg” is the suffix of *pattern*_4,3_ for patterns “abcd” and “abfg.” Therefore, pattern “cfg” can also be not unique.

**Table 2:** Bit-split patterns with two-bit vectors for patterns “abcd,” “abfg,” and “cfg”.

pattern	bit-split pattern	two-bit vectors
abcd	*pattern*_7,0_	01 → 00 → 01 → 00
	*pattern*_6,1_	10 → 11 → 11 → 10
	*pattern*_5,2_	10 → 10 → 10 → 11
	*pattern*_4,3_	00 → 00 → 00 → 00
abfg	*pattern*_7,0_	01 → 00 → 00 → 01
	*pattern*_6,1_	10 → 11 → 11 → 11
	*pattern*_5,2_	10 → 10 → 11 → 11
	*pattern*_4,3_	00 → 00 → 00 → 00
cfg	*pattern*_7,0_	01 → 00 → 01
	*pattern*_6,1_	11 → 11 → 11
	*pattern*_5,2_	10 → 11 → 11
	*pattern*_4,3_	00 → 00 → 00

On the other hand, let us assume that there is a set of patterns “abfh” and “cfg” instead of patterns “abcd,” “abfg,” and “cfg.” As shown in Table 3, no *pattern*_*i*, *j*_ of “cfg” is the suffix of any *pattern*_*i*, *j*_ for “abfh.” Therefore, for the bit-split string matching with two-bit vectors, “abfh” and “cfg” are unique patterns in the example.

**Table 3:** Bit-split patterns with two-bit vectors for patterns “abfh” and “cfg”.

pattern	bit-split pattern	two-bit vectors
abfh	*pattern*_7,0_	01 → 00 → 00 → 00
	*pattern*_6,1_	10 → 11 → 11 → 10
	*pattern*_5,2_	10 → 10 → 11 → 10
	*pattern*_4,3_	00 → 00 → 00 → 01
cfg	*pattern*_7,0_	01 → 00 → 01
	*pattern*_6,1_	11 → 11 → 11
	*pattern*_5,2_	10 → 11 → 11
	*pattern*_4,3_	00 → 00 → 00

## Proposed Parallel String Matching Engine

### Proposed architecture

**Fig 5:**
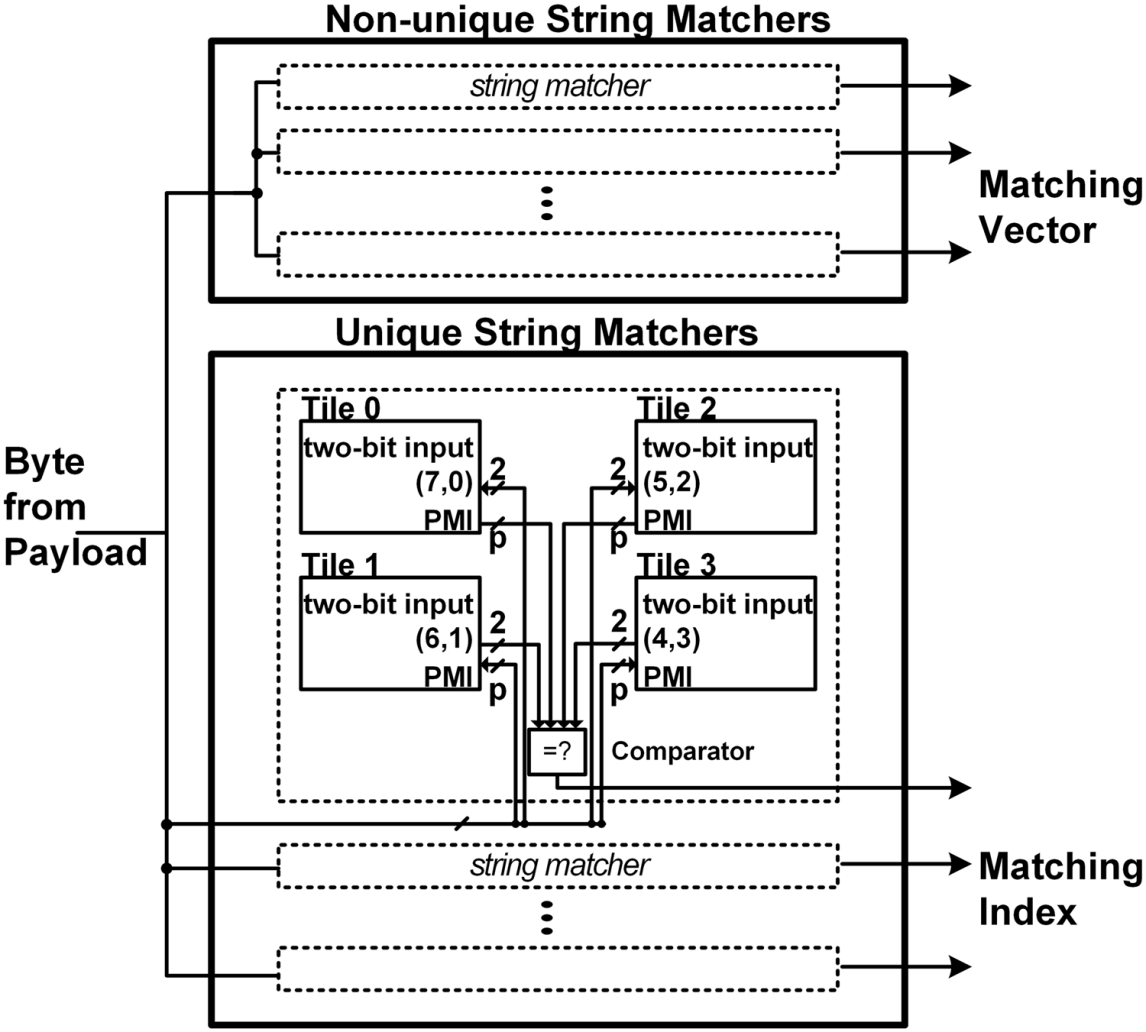
Proposed string matching architecture.

Fig 5 shows the proposed parallel string matching architecture. For the set with non-unique patterns, the traditional bit-split string matchers with PMVs in [8] are applied, where separate PMV tables are adopted in each FSM tile. Considering the previous works in [6], the number of state transitions can be minimized when four DFAs are adopted. Therefore, four FSM tiles have two-bit inputs for each DFA. Considering the balanced inputs for each FSM tile, two bits for an FSM tile input can be selected from both of the MSBs (most significant bits) and the LSBs of the byte input alternately [13].

**Fig 6:**
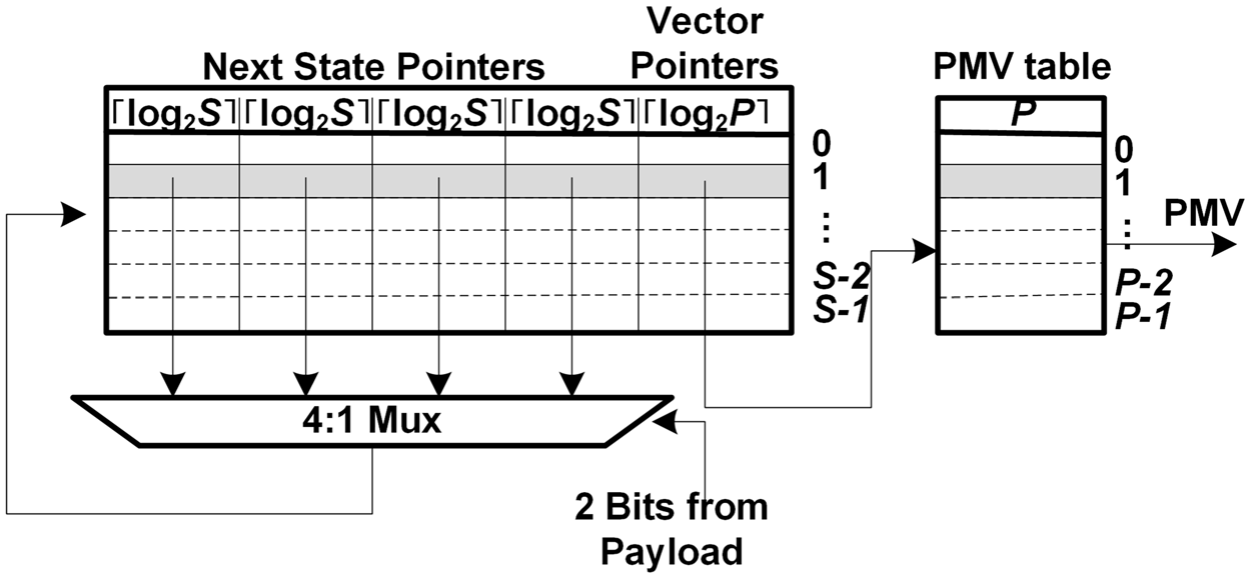
FSM tile in a string matcher for the set with non-unique patterns.

Fig 6 shows the FSM tile in a string matcher for the set with non-unique patterns. Each row represents a state, where the state transitions and vector pointer for the state are stored. With the partial input from the payloads, the next state transition is selected, where the next state transition means the address of the next state in the FSM tile. Parameters *S* and *P* are the maximum numbers of states in an FSM tile and mapped patterns in a string matcher, respectively. The vector pointer in each state indicates its own PMV in the separate PMV table. The numbers in the first row mean the sizes of the state transition, vector pointer, and PMV, respectively.

**Fig 7:**
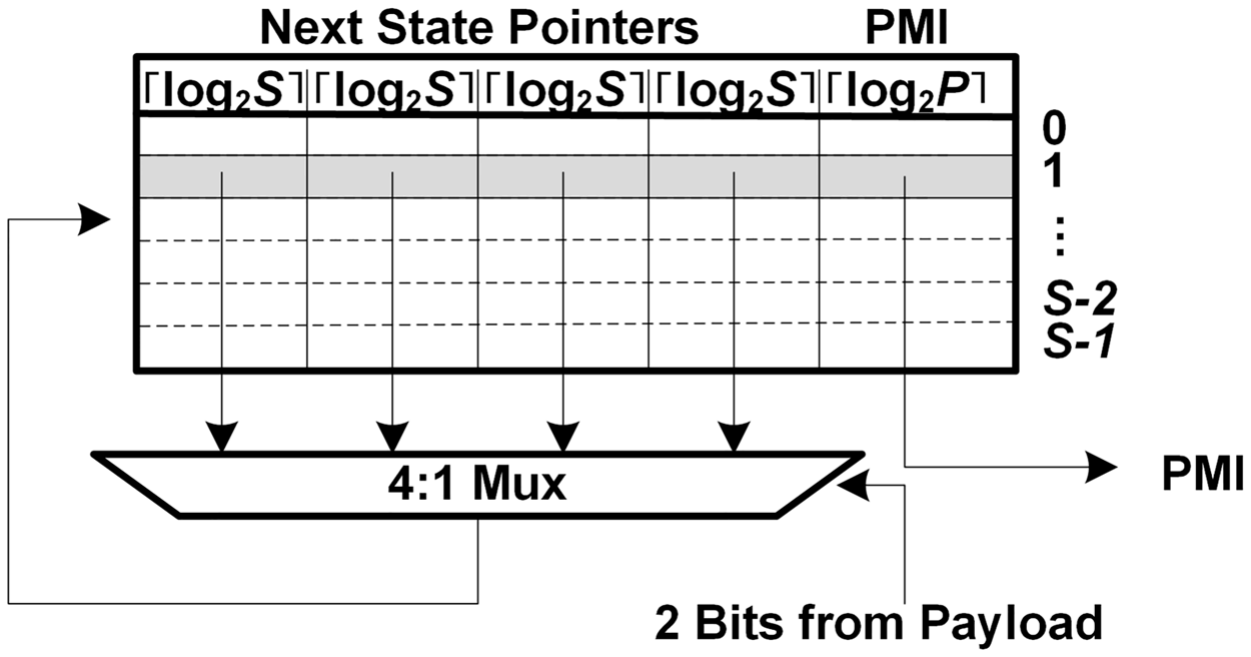
FSM tile in a string matcher for the set of unique patterns.

Fig 7 shows the FSM tile in a string matcher for the set of unique patterns. For the set of unique patterns, unlike previous works in [6, 8–10], the PMVs are not stored; instead, only the unique partial matching index (PMI) for each state is stored in each FSM tile in order to show which pattern is matched. The PMIs from the FSM tiles are compared in order to check whether all of the PMIs are equal or not; if all of the PMIs are the same, the generated matching index indicates its own matched pattern.

### Implementation of proposed string matching engine

Considering the architecture in Fig 5, the proposed string matching engine can be implemented with multiple memory blocks. Therefore, in order to implement the proposed engine with multiple embedded memory blocks, the application specific integrated circuit (ASIC) and FPGA are preferable. Depending on string matcher types, an FSM tile can have different number of memory block cells. In an ASIC, multiple embedded memory macro cells are placed and routed according to semiconductor process. On the other hand, an FPGA can have a fixed number of block memory cells, which are configured into string matchers. In an FPGA such as [26], because the unit size of one block memory cell is predetermined in an FPGA, more than one unit block memory cell can be adopted in an FSM tile. Figs 5, 6 and 7 show that the overhead of combinational logics in the proposed string matching is small. In an ASIC, the combinational logics are implemented using several standard cells. On the other hand, lookup tables (LUTs) are adopted for configuring combinational logics in an FPGA.

A prototype of the original bit-split string matching is shown in [6], where the FSM tile uses block memory cells of an FPGA device. In the bit-split string matching engine in [6], where the memory block of an FSM tile can be configured. In the memory block, both the PMV and next states for a current state exists in a row. Therefore, the PMV and next states can be accessed at the same time.

In the proposed string matching engine, the FSM tile in each non-unique string matcher has a separate PMV table. Therefore, an FSM tile has two separate memory blocks for two tables of state transitions and PMVs. Unlike the original bit-split string matching in [6], the PMV for a state is accessed using the vector pointer in the state transition table. Therefore, the latency for accessing the PMV table is required. However, because the PMV table is separated, the latency does not affect the state transition in a state transition table. In addition, because the time to provide the PMV for a current state can be delayed by one or two cycles regularly, it is expected that there is no critical effect caused by the delayed PMV in terms of performance or throughput. On the other hand, hardware complexity increases due to the separation of memory blocks for storing the PMV table. Considering the combinational logics for accessing the next states and vector pointer, critical path can be related to the multiplexor for state transitions. Because no specific combinational logics are required between vector pointers and PMV table, the increased hardware complexity for the separate PMV table cannot be serious. In each unique string matcher, only one memory block is adopted for implementing an FSM tile. Because separate PMV tables are not needed, there is no increased hardware complexity, compared to the original bit-split string matching.

## Pattern Grouping and Mapping

In the proposed string matching engine, memory contents of FSM tiles are generated by pattern grouping and mapping. After introducing initialization of the string matching engine, details of pattern grouping and mapping are described.

### Initialization of string matching engine

By mapping a rule set of target patterns, the proposed string matching engine is configured, where memory contents for FSM tiles are required. The memory contents are obtained from target patterns using pattern grouping and mapping. After grouping all of the target patterns into the set of unique patterns and set with non-unique patterns, each set is mapped onto multiple string matchers. For the multiple string matchers, the patterns are partitioned into multiple groups. Each partitioned group is mapped onto a string matcher by repeating the pattern mapping for each string matcher. It is noted that the pattern grouping and mapping are not performed in real time. Instead, a tool for the pattern grouping and mapping generates memory contents for target patterns, which are provided for the initialization of string matching engine. The generated memory contents are stored in main memory or on disk. A host processor or controller can manage the initialization with the stored memory contents. In the initialization, memory contents are uploaded in each FSM tile through an interface between host and string matching engine. After uploading the memory contents, string matching can be performed.

### Pattern grouping

In order to map all of the target patterns onto two different types of string matchers, they are grouped into the set of unique patterns and set with non-unique patterns. The pseudo code of the pattern grouping algorithm used for obtaining the set of unique patterns and set with non-unique patterns, *T*_*unique*_ and *T*_*non* − *unique*_, is described in the algorithm in Table 4. Because the probability of matching with multiple short patterns in a state can be high, patterns *T* are sorted in ascending order of their lengths. Then, we check whether pattern *t* is unique among the patterns in a set of patterns *T* using the *Is*_*Unique*(*t*, *T*) function in line 6 of the algorithm in Table 4. In order to speed up the pattern grouping, the *Is*_*Unique*(*t*, *T*) function considers several characteristics. It is assumed that the pattern length of pattern *t*_*i*_ is shorter than or equal to that of pattern *t*_*j*_. By evaluating the *Is*_*Unique*(*t*_*i*_, *T*) function, the uniqueness of pattern *t*_*i*_ is checked against any other patterns including pattern *t*_*j*_. Therefore, after evaluating the *Is*_*Unique*(*t*_*i*_, *T*) function, there is no need to check the pattern uniqueness between *t*_*i*_ and *t*_*j*_ again, which reduces the number of patterns to be checked in the *Is*_*Unique*(*t*_*j*_, *T*) function. The reduction of the number of patterns to be checked is described in line 5 of the algorithm in Table 4. In addition, when applying the *Is*_*Unique*(*t*, *T*) function, if pattern *t* is turned out to be the suffix of any other pattern, the *Is*_*Unique*(*t*, *T*) function does not continue to check the pattern uniqueness against other patterns.

**Table 4:** Proposed pattern grouping algorithm.

1: Sort patterns *T* in ascending order of pattern lengths
2: *T*_*unique*_ ← ∅
3: *T*_*non*–*unique*_ ← ∅
4: **for** each *t* in *T* **do**
5: *T* ← *T* − {*t*}
6: **if** (*Is*_*Unique*(*t*, *T*)) **then**
7: *T*_*unique*_ ← {*T*_*unique*_, {*t*}}
8: **else**
9: *T*_*non* − *unique*_ ← {*T*_*non* − *unique*_, {*t*}}
10: **end if**
11: **end for**
12: Return *T*_*unique*_ and *T*_*non* − *unique*_

On the other hand, from Definition 3, the pattern uniqueness is considered for all sets with the split input symbols. If it is unique, pattern *t* becomes an element in the set of unique patterns *T*_*unique*_; otherwise, pattern *t* becomes an element of the set with non-unique patterns *T*_*non* − *unique*_. By repeating this process, all of the patterns are grouped. Using the obtained two sets, the patterns are mapped onto string matchers.

### Pattern partitioning and mapping

The pattern partitioning algorithm is represented in Table 5 as follows: the set of unique patterns and set with non-unique patterns, *T*_*unique*_ and *T*_*non* − *unique*_, and their string matcher parameters, *M*_*unique*_ and *M*_*non* − *unique*_, are used as the input parameters in the pattern partitioning. There are two main loops for partitioning the patterns in each set. For the set of unique patterns, firstly, all of unique patterns are lexicographically sorted, which increases the probability of there being shared common prefixes in each string matcher. Then, a procedure called *Pattern*_*Mapping*, which denotes the pattern mapping process, is called to obtain the contents of the FSM tiles for a string matcher, *fsms*. In addition, the unmapped patterns, *T*_*u*_, are returned. The FSM tile contents for the string matcher are stored in *vec*_*fsms*_*unique*_. The loop for the set of unique patterns is repeated until there are no more unmapped unique patterns.

**Table 5:** Proposed pattern partitioning algorithm.

1: *T*_*u*_ ← *Lexicographical*_*Sort*(*T*_*unique*_)
2: **while** *T*_*u*_ ≠ *ϕ* **do**
3: *fsms*, *T*_*u*_ ← *Pattern*_*Mapping*(*T*_*u*_, *M*_*unique*_)
4: *vec*_*fsms*_*unique*_ = *vec*_*fsms*_*unique*_ + *fsms*
5: **end while**
6: *T*_*u*_ ← *Lexicographical*_*Sort*(*T*_*non* − *unique*_)
7: **while** *T*_*u*_ ≠ *ϕ* **do**
8: *fsms*, *T*_*u*_ ← *Pattern*_*Mapping*(*T*_*u*_, *M*_*non* − *unique*_)
9: *vec*_*fsms*_*non* − *unique*_ = *vec*_*fsms*_*non* − *unique*_ + *fsms*
10: **end while**
11: Return *vec*_*fsms*_*unique*_ and *vec*_*fsms*_*non* − *unique*_

The second loop is repeated in order to obtain the contents of the FSM tiles for the set with non-unique patterns. The process for the next loop is similar to that of the first loop, as shown in the algorithm of Table 5. Finally, the FSM tile contents, *vec*_*fsms*_*unique*_ and *vec*_*fsms*_*non* − *unique*_ are returned for the adopted multiple string matchers.

The procedure of pattern mapping, *Pattern*_*Mapping*, can generate the contents of the FSM tiles for a string matcher based on the lexicographical sorting in [6]. In the pattern mapping, several hardware resource limitations such as the maximum numbers of patterns to be mapped, *P*, and states, *S*, are considered in the pattern mapping. The pattern mapping maximizes the number of patterns mapped onto a string matcher as follows: Firstly, the list of the sorted patterns is provided as the input of this procedure. The contents for the FSM tiles are built with the front *k* patterns determined from the inputted list. In the first iteration, *k* is set to *P*. If the required number of states among the obtained DFAs for the FSM tiles is greater than that available in a homogeneous FSM tile, *S*, the patterns could not be mapped onto the string matcher. In this case, after decreasing *k* by one, the process mentioned above is iterated until the required number of states in each FSM tile is smaller than *S*. After the iteration is completed, the failing pointer addition is performed. Then, the unmapped target patterns and contents of the FSM tiles for the string matcher are returned.

### Algorithm complexity of pattern grouping and mapping

In the pattern grouping algorithm, the uniqueness of each pattern is checked against all of the target patterns. Firstly, in the pattern grouping, all patterns are sorted in ascending order of their lengths. In this case, the time complexity of pattern sorting with pattern lengths is *O*(*N* ⋅ *log*_2_ *N*), where *N* is the number of all target patterns. After sorting *N* patterns, patterns are indexed by *t*_1_, *t*_2_,…, *t*_*N* − 1_, *t*_*N*_. In the evaluation of the *Is*_*Unique*(*t*_1_, *T*) function for the shortest pattern, the pattern uniqueness is evaluated with *N* − 1 pairs. On the other hand, the evaluation of the *Is*_*Unique*(*t*_*N* − 1_, *T*) function checks the pattern uniqueness with one pair of *t*_*N* − 1_ and *t*_*N*_. In addition, there is no pair to be checked in the evaluation of the *Is*_*Unique*(*t*_*N*_, *T*) function. Therefore, the time complexity of this process is $O(\frac{N(N-1)}{2})=O(N^{2})$. Considering the pattern sorting and repeated evaluations of the *Is*_*Unique*(*t*, *T*) function, the time complexity of the pattern grouping can be *O*(*N* ^2^).

After grouping all of target patterns into the set of unique patterns and set with non-unique patterns, these two sets are used as the input parameters for the pattern partitioning. Due to the hardware resource parameters, the numbers of mapped patterns and states in each FSM tile are limited. Therefore, the pattern mapping for a string matcher shows constant time complexity, *O*(1). In addition, the complexity of the **while** loop in the pattern mapping can be *O*(*N*) because the entire set of patterns is mapped onto multiple string matchers. Therefore, when the maximum length of patterns is limited, the time complexity for partitioning the entire set of patterns can be *O*(*N*). On the other hand, the time complexity of lexicographical sorting in the pattern partitioning algorithm can be *O*(*N* ⋅ *log*_2_ *N*). However, because there are large constant factors in the pattern mapping, if the number of target patterns is not sufficiently large, the pattern sorting will not be a dominant factor in the time complexity. Therefore, it can be concluded that the time consumed for obtaining the contents of FSM tiles is proportional to *N*.

## Experimental Results and Discussion

### Experimental environments

The proposed pattern matching scheme was evaluated using a C++ library. The pattern grouping and mapping were performed using a machine with Intel Xeon E31270 CPU, 8 Gbytes main memory, and CentOS 6.5 Linux operating system [27]. Four sets of target patterns denoted as *backdoor*, *deleted*, *spyware*, and *web-client* were extracted from the Snort v2.8 rules [11]. For the evaluation of the set with many patterns, a set of total patterns denoted as *total* was extracted from all of the Snort v2.8 rules [11]. In addition, a rule set from the ClamAV 0.95.3 [12] denoted as *clamAV* was adopted.

Table 6 shows the characteristics of the target pattern rule sets, where *num*(*patterns*) and *num*(*bytes*) mean the number of target patterns and total sum of characters of each rule set, respectively. In addition, max(*l*) and avg(*l*) are the maximum pattern length and average pattern length, respectively. The column in the rightmost (*σ*) describes the standard deviation of pattern lengths.

**Table 6:** Characteristics of target pattern rule sets.

rule name	num(patterns)	num(bytes)	max(*l*)	avg(*l*)	*σ*
*backdoor*	955	8,875	94	9.3	7.5
*deleted*	615	7,399	72	12.0	11.0
*spyware*	2,299	26,103	94	11.4	8.1
*web-client*	1,657	67,527	92	40.8	22.8
*total*	7,784	144,958	122	18.6	18.0
*clamAV*	28,786	1,921,305	210	63.2	40.8

For the apples-to-apples comparisons, the lexicographical pattern mapping in [6], which has been used for other string matching schemes, was adopted. Based on the design analysis for the bit-split string matching in [6], each FSM tile took a two-bit input. In order to ensure that the implementation was realistic, the unit size of memory cells was considered. In the experiments, 1-Kbit, 2-Kbit, 4-Kbit, and 8-Kbit unit sizes of block memory cells were assumed. These assumed memory sizes can be found in commercial FPGA devices. For example, considering the block memory in the Cyclone II of Altera [26], the unit size of each block memory cell was assumed to be 4 Kbits. The memory requirements can be calculated using the number of required memory cells. Several parameters were swept to obtain the optimal parameter values with the minimum number of memory blocks. For the Snort rule sets, the maximum number of states in an FSM tile, *S*, was 128 or 256. The maximum number of mapped patterns in a string matcher *P* was 16, 32, 48, or 64 when *S* was 128. When *S* was 256, *P* was 32, 64, 96, or 128. On the other hand, for the ClamAV rule set, considering the maximum target pattern length of 210, only 256 was adopted for *S*.

### Experiments with Snort and ClamAV rule sets

The evaluations were performed taking into consideration the experimental environments mentioned above. In the experiments with the Snort rule sets, the numbers of memory cells were minimized when *S* was 128. For the ClamAV rule set, the experiments were performed when *S* was 256. Therefore, Table 7 shows the required number of memory blocks obtained by sweeping the parameters for a string matcher when *S* was 128 and 256. The data shown in Table 7 proves that the string matcher for the set of unique patterns can reduce the memory requirements, compared to that for the set with non-unique patterns. In addition, the memory requirements did not increase proportionally to *P*, which proves that the memory requirements of the unique PMIs were small.

**Table 7:** Required numbers of memory cells according to hardware limitations and unit sizes of memory cells.

		*P*_*non* − *unique*_				*P*_*unique*_			
*S*	*Mem*	16	32	48	64	16	32	48	64
128	1 Kbits	20	24	32	36	16	20	20	20
	2 Kbits	12	16	20	20	8	12	12	12
	4 Kbits	8	12	12	12	4	8	8	8
	8 Kbits	8	8	8	8	4	4	4	4
*S*	*Mem*	32	64	96	128	32	64	96	128
256	1 Kbits	44	56	76	104	40	40	40	40
	2 Kbits	24	28	40	52	20	20	20	20
	4 Kbits	16	16	24	28	12	12	12	12
	8 Kbits	12	12	16	16	8	8	8	8

According to the algorithm in Table 4, the set with non-unique patterns and set of unique patterns were obtained. Table 8 shows the numbers of patterns in the two sets for the six rule sets. The ratio of unique patterns to all of target patterns in a rule set was from 32.7% to 95.4%. Due to the long average pattern lengths of *web-client* and *clamAV*, the ratio of unique patterns was high. Considering the experimental results in Table 8, minimizing the memory requirements using the set of unique patterns could be an effective strategy. Compared to the previous bit-split string matching, additional time was required in the pattern grouping. The pattern grouping for all Snort rule sets was finished within 1 second. For the ClamAV rule set, due to the large number of target patterns, the time to be required in the pattern grouping was 15.7 seconds. Considering the large number of patterns in the ClamAV rule set, even though the complexity of the pattern grouping algorithm was *O*(*N* ^2^) for a set with *N* patterns, the required time for processing the pattern grouping was not great.

**Table 8:** Numbers of patterns in the set with non-unique patterns and set of unique patterns.

set	#non-unique patterns	#unique patterns	ratio (unique/total)	*T*_*grouping*_ (sec)
*backdoor*	643	312	32.7%	0.01
*deleted*	334	281	45.7%	0.01
*spyware*	1,476	823	35.8%	0.02
*web-client*	370	1,287	77.7%	0.06
*total*	4,446	3,338	42.9%	0.28
*clamAV*	1,324	27,462	95.4%	15.7

Table 9 lists the minimized memory requirements with the maximum number of patterns to be mapped, *P*, and number of string matchers for different unit sizes of memory cells. When the unit size of a memory cell was 1 Kbits, the memory requirements were minimized because unused memory bits were the smallest. For the set of unique patterns in the Snort and ClamAV rule sets, the memory requirements were minimized when *P* was 16 and 32, respectively. Considering the process of DFA construction, as the length of a target pattern increased, the probability of its being the unique pattern decreased. Therefore, compared with the average pattern length for the set with non-unique patterns, the average pattern length for the set of the unique patterns was longer. For the set with non-unique patterns of *web-client* and *clamAV*, due to the long average pattern length, the memory requirements were minimized when *P* was 16 and 32, respectively. On the other hand, a *P* value of 32 or 64 was adopted for the sets with the non-unique patterns in the other Snort rule sets because of the short average pattern lengths.

**Table 9:** Total memory requirements according to different unit sizes of memory cells.

1 Kbits	non-unique		unique		total memory
	#matchers	*P*	#matchers	*P*	
*backdoor*	21	32	35	16	136 KB
*deleted*	38	32	11	16	112 KB
*spyware*	51	32	106	16	374 KB
*web-client*	91	16	642	16	1,547 KB
*total*	228	32	928	16	2,601 KB
*clamAV*	160	32	8,671	32	45,297 KB
2 Kbits	non-unique		unique		total memory
	#matchers	*P*	#matchers	*P*	
*backdoor*	15	64	35	16	149 KB
*deleted*	8	64	38	16	119 KB
*spyware*	51	32	106	16	426 KB
*web-client*	91	16	642	16	1,594 KB
*total*	228	32	928	16	2,834 KB
*clamAV*	160	32	8,671	32	45,379 KB
4 Kbits	non-unique		unique		total memory
	#matchers	*P*	#matchers	*P*	
*backdoor*	15	64	35	16	164 KB
*deleted*	8	64	38	16	127 KB
*spyware*	42	64	106	16	475 KB
*web-client*	91	16	642	16	1,688 KB
*total*	208	64	928	16	3,179 KB
*clamAV*	160	32	8,671	32	54,585 KB
8 Kbits	non-unique		unique		total memory
	#matchers	*P*	#matchers	*P*	
*backdoor*	15	64	35	16	266 KB
*deleted*	8	64	38	16	221 KB
*spyware*	42	64	106	16	778 KB
*web-client*	91	16	642	16	3,326 KB
*total*	208	64	928	16	5,505 KB
*clamAV*	160	32	8,671	32	72,999 KB

**Fig 8:**
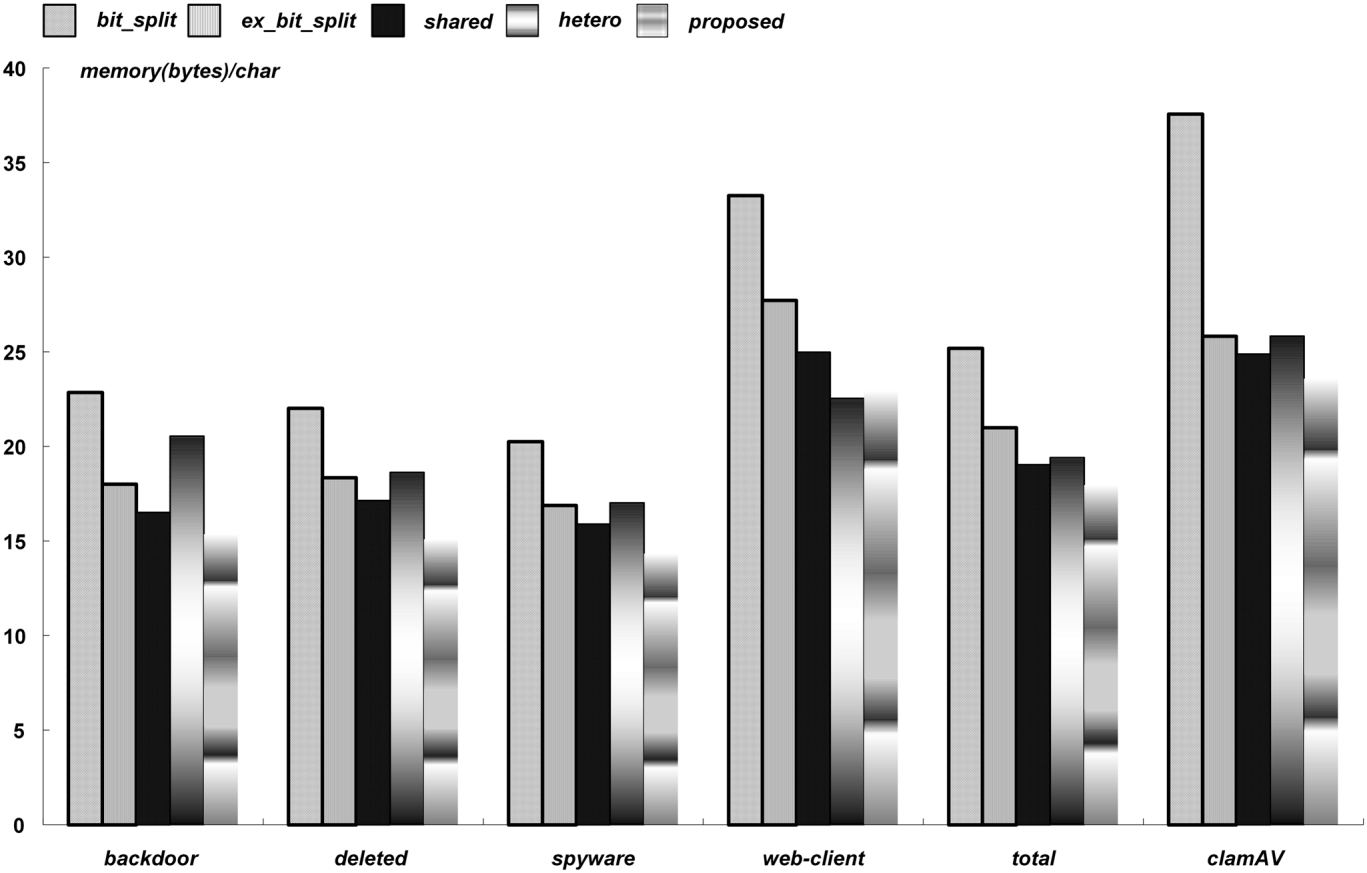
Summary of comparisons for 1-Kbit memory cells.

**Fig 9:**
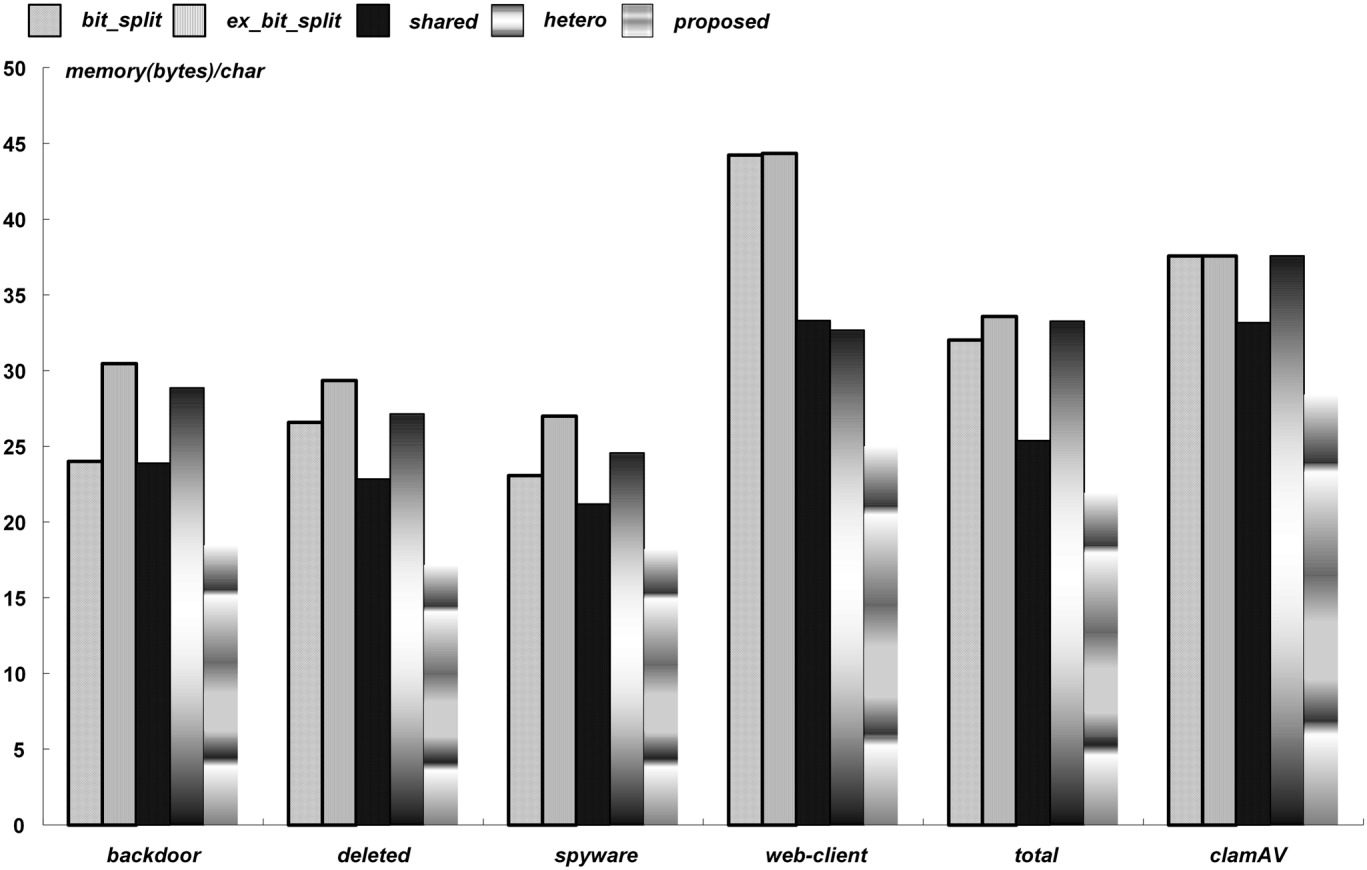
Summary of comparisons for 4-Kbit memory cells.

Figs 8 and 9 provide a summary of the comparisons with the existing bit-split string matching approaches in terms of the normalized memory requirements for the cases using 1-Kbit and 4-Kbit memory cells, respectively. The normalized memory requirements of a rule set were obtained after dividing the minimized memory requirements by the total sum of bytes in the target patterns of a rule set. The unit of the normalized memory requirements, therefore, was *bytes/char*.

For the six rule sets, the normalized memory requirements ranged from 14.3 to 23.6 (bytes/char) with 1-Kbit memory cells. On the other hand, the normalized memory requirements ranged from 17.2 to 28.4 (bytes/char) with 4-Kbit memory cells. Because unused memory bits were increased with the unit size of memory cells, the normalized memory requirements with 4-Kbit memory cells increased. In the *web-client* and *clamAV*, because the average pattern length was long, the numbers of mapped patterns in a string matcher were small, which increased the total memory requirements.

The string matching schemes in [6, 8–10], were denoted as *bit_split*, *ex_bit_split*, *shared*, and *hetero*, respectively. With 1-Kbit memory cells, the total memory requirements were decreased on average by 31.8%, 14.7%, 8.0%, and 12.4%, compared with *bit_split*, *ex_bit_split*, *shared*, and *hetero*, respectively, On the other hand, with 4-Kbit memory cells, the total memory requirements were decreased on average by 29.8%, 36.0%, 19.1%, and 30.1%. Therefore, the proposed string matching approach was more efficient when a large unit size of memory cells was adopted. Considering the summary in Figs 8 and 9, it was concluded that the proposed scheme can significantly reduce the memory requirements for the bit-split string matching architecture.

### Analysis of pattern uniqueness

For more structural and statistical analysis, several rule sets with random patterns were generated. In this case, evaluations were performed by sweeping several parameters of the average pattern length, standard deviation, and number of target patterns. In the evaluations of the average pattern lengths and numbers of target patterns, considering the characteristics of real patterns shown in Table 6, it was assumed that the average pattern length was the same as the standard deviation of pattern lengths. Considering experimental data for the realistic rule sets mentioned above, it was concluded that the memory requirements for the set of unique patterns were smaller than those of the set with non-unique patterns in a string matcher. Therefore, after several rule sets were generated, the ratio of unique patterns in each rule set was evaluated in order to analyze the pattern uniqueness in a rule set.

**Fig 10:**
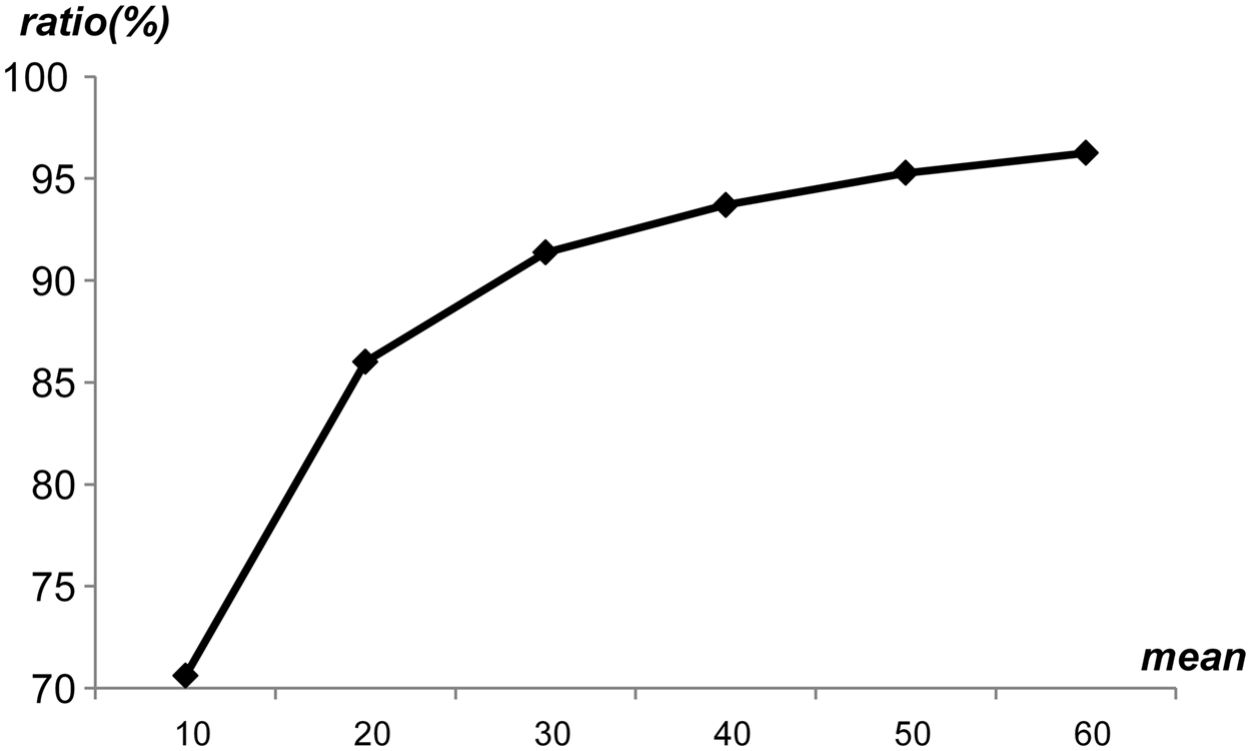
Ratio of unique patterns by sweeping the average pattern length.

**Fig 11:**
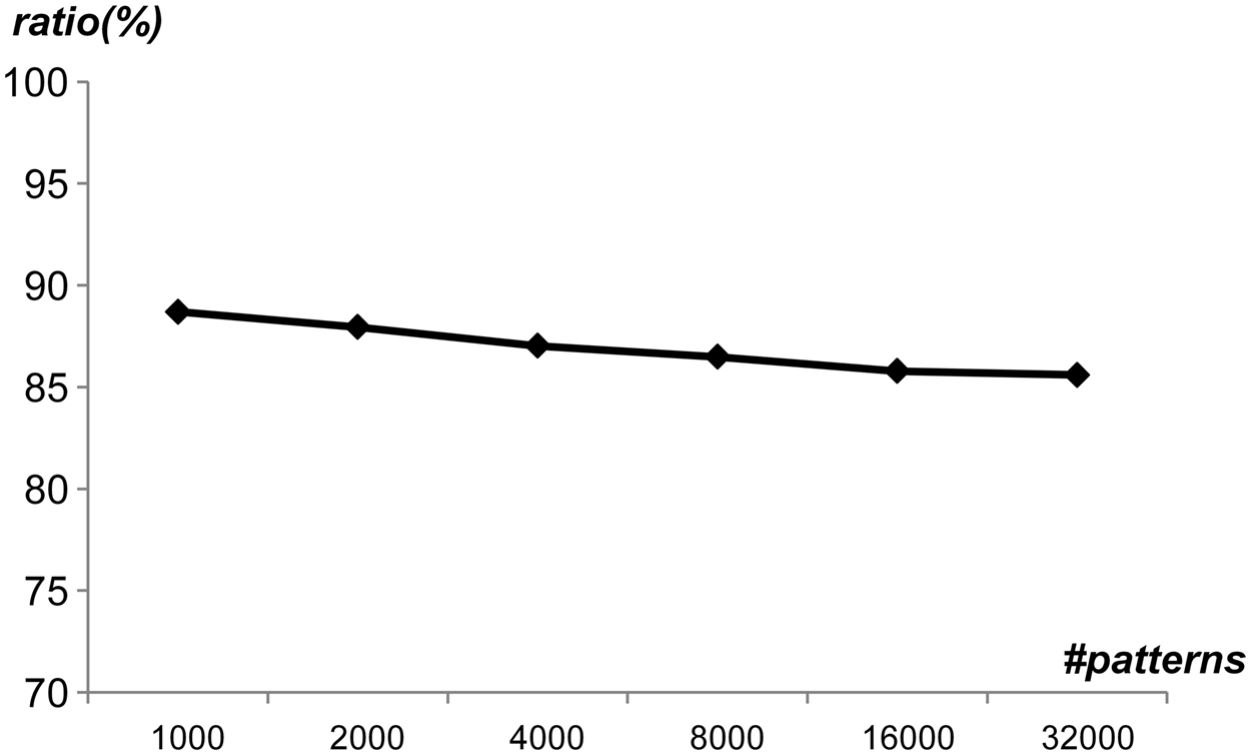
Ratio of unique patterns by sweeping the number of generated patterns.

**Fig 12:**
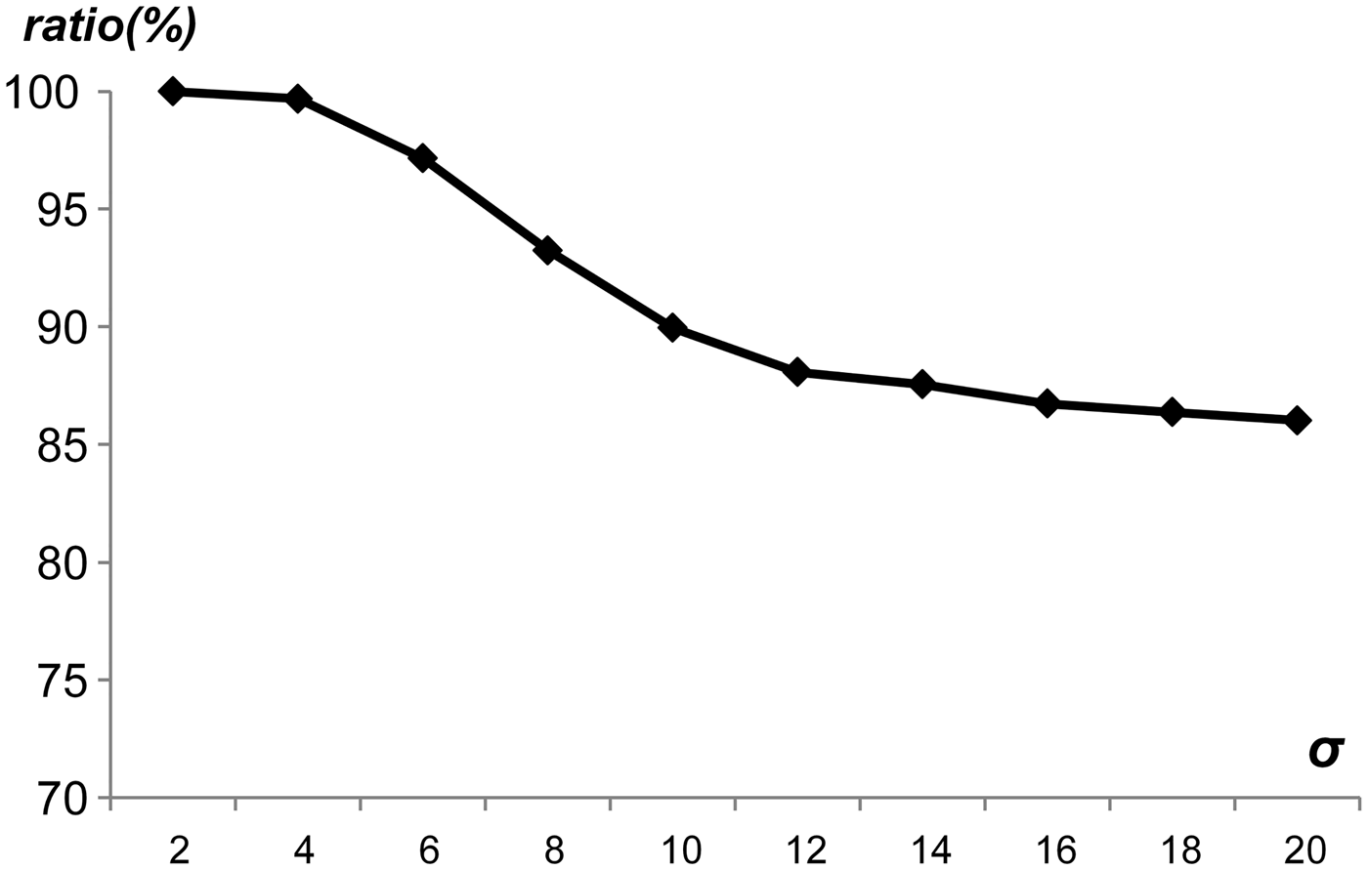
Ratio of unique patterns by sweeping the standard deviation.

Firstly, the average pattern length was swept from 10 to 60 with several generated rule sets, where each rule set had 10,000 different patterns. Fig 10 shows the ratio of unique patterns by sweeping the average pattern length, where the average pattern length was denoted as *mean*. As shown in Fig 10, the ratio of unique patterns was 70.6% when *mean* was 10. When *mean* was 60, the ratio of unique patterns reached up to 96.3%. Therefore, as *mean* increased, the ratio of unique patterns increased. In addition, even though the average pattern length was short, the ratio of unique patterns was over 70%, which means that the proposed string matching scheme can utilize the pattern uniqueness to reduce the memory requirements without the PMV table. Fig 11 shows the ratio of unique patterns by sweeping the number of generated patterns from 1,000 to 32,000. In this case, it was assumed that the average pattern length was 20. As the number of patterns in a rule set increased, the ratio of unique patterns decreased slightly. In addition, 86% percent of patterns were in the set of unique patterns when the number of patterns was 32,000. Fig 12 shows the ratio of unique patterns by sweeping the standard deviation from 2 to 20, where the average pattern length was 20. When the standard deviation was small, the ratio of unique patterns was high. As the standard deviation increased, the ratio of unique patterns decreased slightly. However, the ratio of unique patterns was over 86% when the standard deviation was 20. Considering the experimental data for the pattern uniqueness, it was expected that the ratio of unique patterns can be high in a general rule set, which can reduce the memory requirements with the proposed scheme.

### Practical implementation

In order to know the hardware overhead of the proposed string matching engine, the string matcher was coded using Verilog hardware description language (HDL). Then, the code was compiled using Altera’s Quartus II, where Cyclone II EP2C70F89618 [26] was selected for the target device. Even though there were several high-performance FPGA device families, the overall price was too high. Therefore, the Cyclone II FPGA family was selected for the general string matching engine. In the target device, there were 68,416 logic elements and registers. In addition, 250 M4K memory cells can be configured. In a M4K memory cell, 4,096 memory bits were contained. In the implementations, several pairs of the maximum numbers of states and patterns to be mapped, which was denoted as (*S*, *P*), were adopted.

Table 10 shows the implementation results of the unique and non-unique string matchers, where four sets of (*S*, *P*) were evaluated. The separation of memory blocks in each FSM tile of the non-unique string matcher increased the routing complexity. Therefore, in Table 10, the maximum operating frequency, *F*_*max*_, of the unique string matcher was higher than that of the non-unique string matcher. In each clock cycle, one ASCII character can be inputted. Considering the critical path in the non-unique string matcher, the throughput can reach up to 1.127 Gbps (Giga bits per second) when (*S*, *P*) was (128, 16) in Table 10. As *S* increased, *F*_*max*_ decreased gradually due to the increasing complexity in memory blocks. However, the decreased value of *F*_*max*_ was not proportional to *S*. Compared to the memory resource usage, the ratio of used logic elements and registers was very low. Therefore, it was concluded that the memory resource was more critical in the implementation of the string matching engine. In addition, small *S* can be better for efficient hardware resource usage and high throughput. In order to update the memory contents in memory blocks, several logic elements and registers were required in each memory block. In the non-unique string matcher, because there were two separate memory blocks, more logic elements and registers were required compared to those of the unique string matcher. Considering the implementation results mentioned above, by adopting the unique string matchers, it was expected that the hardware overhead can be reduced.

**Table 10:** Implementation results of a string matcher according to the maximum numbers of states and patterns to be mapped.

unique string matcher				
(*S*, *P*)	(128,16)	(256,32)	(512,64)	(1024,128)
logic elements	237	274	311	348
registers	44	52	60	68
M4K memory cells	4	12	20	48
*F*_*max*_ (MHz)	156.6	155.9	154.2	140.7
non-unique string matcher				
(*S*, *P*)	(128,16)	(256,32)	(512,64)	(1024,128)
logic elements	381	561	885	1,497
registers	92	160	292	552
M4K memory cells	8	16	28	64
*F*_*max*_ (MHz)	140.9	133.8	132	114.5

## Conclusions

The proposed string matching scheme can be applied to memory-based bit-split string matching, where the memory requirements can be reduced by eliminating the matching vectors for the set of unique patterns. The proposed pattern grouping is used in order to obtain the set of unique patterns. In addition, the pattern partitioning and mapping algorithms are adopted for the parallel string matching engine. Considering the experimental results with various rule sets, the proposed string matching scheme is greatly helpful for reducing the storage cost with the regularity and scalability of the bit-split parallel string matching engine.
